# Effect of Filler Size and Temperature on Packing Stress and Viscosity of Resin-composites

**DOI:** 10.3390/ijms12085330

**Published:** 2011-08-18

**Authors:** Haitham Elbishari, Julian Satterthwaite, Nick Silikas

**Affiliations:** School of Dentistry, University of Manchester, Higher Cambridge Street, Manchester M15 6FH, UK; E-Mails: julian.satterthwaite@manchester.ac.uk (J.S.); nick.silikas@manchester.ac.uk (N.S.)

**Keywords:** packing, resin-composites, nanofillers, viscosity

## Abstract

The objective of this study was to investigate the effect of filler size on the packing stress and viscosity of uncured resin-composite at 23 °C and 37 °C. A precision instrument used was designed upon the penetrometer principle. Eight resin-composite materials were tested. Packing-stress ranged from 2.60 to 0.43 MPa and viscosity ranged from 2.88 to 0.02 MPa.s at 23 °C. Values for both properties were reduced significantly at 37 °C. Statistical analysis, by ANOVA and *post hoc* methods, were carried out to check any significant differences between materials tested (P < 0.05). Conclusions: Filler size and distribution will affect the viscosity and packing of resin-composites during cavity placement.

## 1. Introduction

The demand for dental aesthetic restorations has led to the development of resin-composite material. Early resin-composites gave rise to concerns regarding toughness, durability and strength [1].

Typically, dental composites consist of a matrix and fillers bound together. Several improvements of these two components over the last 20 years have increased the use of dental composites and in many cases have replaced amalgam as the restorative material [2,3]. Despite all improvements in dental composites, fracture of restorations, particularly in large cavities in the posterior region, is one of the most common causes of resin-composite restoration failure for the first five years of placement and the second most common cause of failure between five and ten years of placement [4–7]. In order to address this, efforts have been focussing on either altering the monomer system or improving filler technology and the use of fibres to reinforce the matrix [8,9]. Recently, polymer nanofibres and titania nanoparticles have been added to resin composite to improve its properties [10]. Current composite materials are almost as strong and tough as amalgam, but not as strong as ceramic and casting alloys [11]. However, these improvements in their mechanical properties have affected the viscosity of resin composite [12,13]. Their viscosity is directly related to ease of resin placement, malleability and stickiness to tooth and instruments in so called handling characteristics [14–18].

While the effect of resin composite filler size and shape on the mechanical properties [19,20] and shrinkage [21] have been documented in the literature, the effect of filler size and morphology on the rheological behaviour of uncured resin composite is minimal [22,23].

Therefore, the aim of this study was to investigate the effect of different filler size and distribution on the packing stress and viscosity of uncured resin composites at two different temperatures (23 °C *versus* 37 °C). The null hypotheses were that different filler size, distribution and temperature have no effect on: (i) the packing stress; and (ii) viscosity of uncured resin composite.

## 2. Results and Discussions

**Figure 1.**
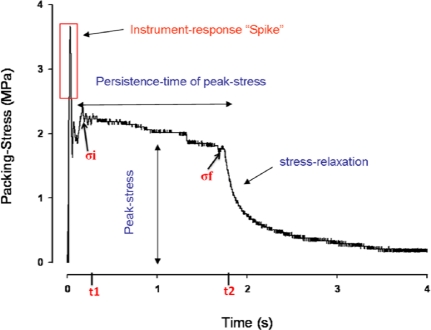
Time dependant packing stress profile curve.

Advanced developments in filler technology of resin composites have steered the improvement process of optimizing resin composite properties. This study aimed to investigate the effect of different filler sizes and distributions on the handling properties of resin composites at both clinic temperature (23 °C) and patient body temperature (37 °C). Packing stress and viscosity were investigated for different resin-composites that range in filler size from 100–1500 nm; and vary in filler distribution (*i.e.*, uni-modal, bi-modal and tri-modal). The packing stress was measured by the load cell as illustrated by the stress-time curve shown in Figure 1. Means and standard deviations of both packing stress and viscosity are presented in Tables 1 and 2. Statistically significant differences were present among each property tested (P < 0.05) at both temperatures as shown in the tables. Accordingly, both null hypotheses were rejected.

**Figure 2.**
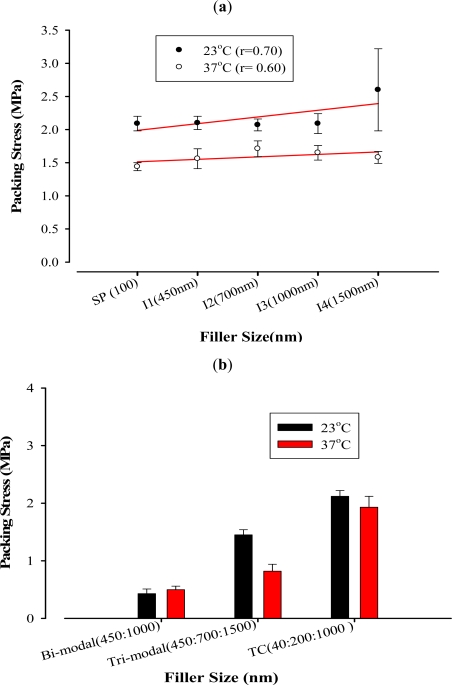
Effect of varying filler sizes at 23 °C and 37 °C on packing stress (MPa): (**a**) Linear correlations between packing stress (MPa) and unimodal composites at 23 °C and 37 °C; and (**b**) Bar Chart of packing stress (MPa) at 23 °C and 37 °C for multimodal composites.

**Figure 3.**
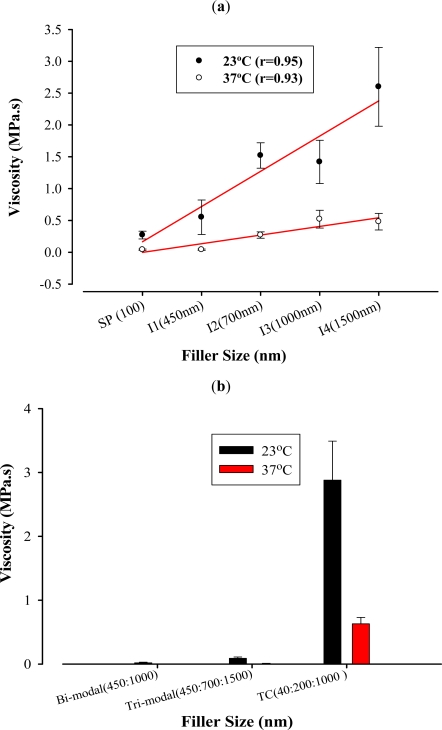
Effect of varying filler sizes at 23 °C and 37 °C on viscosity (MPa.s): (**a**) Linear correlations between viscosity (MPa.s) and unimodal composites at 23 °C and 37 °C; and (**b**) Bar Chart of viscosity (MPa.s) at 23 °C and 37 °C for multimodal composites.

Generally, as the fillers increased in size, packing stress and viscosity increased at both temperatures. Positive correlation was evident as shown in Figures 2(a) and 3(a). However, this increase was not statistically significant among some filler sizes (P > 0.05).

Uni-modal composites showed the same trend with packing stress and viscosity at both temperatures. Filler size of 1500 nm exhibited the highest packing stress at 23 °C (2.60 MPa). Despite the positive correlation between filler size and packing stress at 23 °C and 37 °C (r = 0.70 and 0.60 respectively) and between filler size and viscosity 23 °C and 37 °C (r = 0.95 and 0.93 respectively), the increase in both packing stress and viscosity was not statistically significant amongst most unimodal composites. This can clearly be seen between SP (100 nm) and I1 (450 nm) which could be due to the difference in filler shape.

The trend of multimodal composites was the same with packing stress and viscosity at both temperatures. Tetric Ceram, which has three different filler sizes (40:200:1000 nm) in so called tri-modal fillers, exhibited not only the highest values among multimodal composites, but also was the highest among all materials in packing stress at 37 °C and viscosity at both temperatures. This material was also the second highest in packing stress at 23 °C (P < 0.05). This is probably due to the fact that resin-composite material achieves its thicker consistency by increasing filler size, modifying filler distribution and adding other types of fillers such as glass fibres [24]. Moreover, as the temperature increases the flow of the resin composite increases as the resin matrix becomes diluted [25].

Tetric Ceram was the most viscous material at both temperatures among all the materials tested (P < 0.05) which could be due to the higher volume and weight percentage of filler content. However, there was a significant reduction in its viscosity when tested at 37 °C, and this is likely due to the fact that an increase in temperature decreases the viscosity [26].

On the other hand, the I5 (a bimodal 450:1000) exhibited the lowest viscosity among all materials. Its viscosity was remarkably low, despite the fact that the two different filler sizes were identical to the unimodal I1 and I3 respectively, that exhibited higher viscosity values. Furthermore, its packing stress was also the lowest. The tri-modal I6 (450:700:1500, 1:1:3) also presented lower viscosity values compared to the unimodal formulations. It appears that the combination of filler sizes result in more flowable and less stiff composites.

Within the limitations of this study, it can be concluded that:

1. Filler size and distribution have an effect on the packing stress and viscosity.
2. Temperature has a prominent effect on the handling properties of resin-composite, *i.e.*, as temperature increases the packing stress and viscosity decreases.
3. Filler sizes and their combinations (bimodal and trimodal distributions) can have a fine-tuning effect on the handling properties and clinical performance.

## 3. Materials and Methods

The resin-composites used in the study were all visible light cured, and included 7 model formulations (Ivoclar Vivadent, Schaan, Liechtenstein) together with an established commercially available formulation (Tertic Ceram [TC]-Ivoclar Vivadent, Schaan, Liechtenstein) used as a control.

The resin matrix was the same for all materials and was a combination of BisGMA, UDMA and TEGDMA with 0.33% camphoroquinone. All of the model composites had a particulate dispersed phase of the same volume fraction (56.7%), which was treated with a silane coupling agent (methacryloxypropyltrimethoxysilane). The filler particles were systematically graded in size, and were either spherical or irregular in shape. The spherical particles were silica, and the irregular particles were ground glass (Ba-Al-B-silicate glass).

Tetric Ceram contained heterogeneous, multimodal filler particles, comprising Barium glass 1 μm, Ba-Al-FB-silicate 1 μm, SiO_2_ 40 nm, spherical mixed oxide 0.2 μm, and ytterbium trifluoride. The composition of the resin-composites is summarized in Table 3.

**Figure 4.**
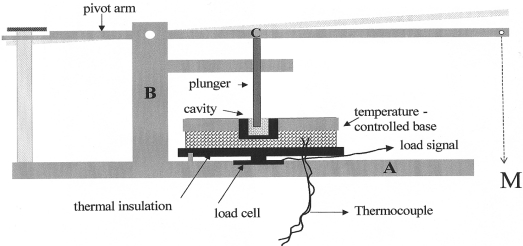
Schematic diagram showing various parts of the packing stress measurement apparatus: A—steel base; B—steel pillar; C—friction bearing; D—temperature controlled base; M-weight.

A precision instrument was designed and fabricated upon the penetrometer principle. The apparatus used (Figure 4) consisted of a lever with an arm pivoting via a load-bearing pin, on a vertical steel pillar B bolted to a steel base A. The lever, pillar and steel base formed a horizontal U shape with the lever extending beyond the base. A thin cylindrical rod (diameter = 3 mm) was pushed via the lever arm into each unset material to a controlled depth (2.5 mm) under a constant load.

The control of penetration depth was achieved by a stop plate mounted on an additional pillar. A reduced friction bearing C was also vertically positioned to limit any angular motion of the lever produced a linear displacement of the rod. The test samples were placed in a movable cavity within the temperature controlled base D. A calibrated thermocouple tip inserted into a hole drilled into the rim of temperature controlled base monitoring the temperature of the cavity, when connected to electrical supply. The free end of the lever was weighted by a 500 g mass M.

A movable open ended cylindrical brass small cavity (6.35 mm diameter and 4.5 mm depth) with two different controlled temperatures used in this study.

142.44 mm^3^ of composite material was placed in the cavity using a flat end plastic hand instrument, a glass slab used to level the material with the mould’s surface. The plunger flat end was placed lightly on the surface of the composite material to be investigated, following the first test, composite material repacked into the mould, adding material also done as required, plunger head cleaned and test repeated six times (n = 6) for each material. A representative recording of stress is shown in Figure 1: upon application of the plunger, an initial “spike” in stress is recorded: the “persistence time of peak stress” [tp] was taken as the time after the initial spike [t1] to the time of dissipation of recorded stress [t2]. The ‘mean packing stress’ [σ] was taken as the average of the stress recorded at t1 [σi] and t2 [σf]. The viscosity [µ] was calculated (Equation 1) as the mean packing stress multiplied by the persistence time of peak stress, thus:

(1) $$\mu =\mathrm{tp}\left(\frac{\sigma i+\sigma f}{2}\right)$$

All materials were investigated at two temperatures 23 °C and 37 °C.

Packing stress and viscosity data among the eight groups were analysed using One-Way ANOVA (v. 16, SPSS, Il, USA) (P < 0.05) Prior to *post hoc* tests, data were analysed for equal variances using homogeneity test (P < 0.05). For data of packing stress at 37 °C, and viscosity measurements at both temperatures, equal variances can be assumed, thus Bonferroni test was applied; however Dunnett’s T3 was applied for data of packing stress at 23 °C as equal variances cannot be assumed). Effect of the temperature on each material was analysed using t-test for paired data (P < 0.05). Linear correlation was checked between filler size and packing stress at both temperatures and between filler size and viscosity at both temperatures.

## Figures and Tables

**Table 1.** Mean (SD) values of packing stress (MPa) of different resin composites at 23 °C and 37 °C.

**Group**	**Packing Stress at 23 °C Mean (**SD**)**	**Packing Stress at 37 °C Mean (**SD**)**
**I1**	**2.10** (0.10) ^a*^	**1.56** (0.15) ^a,b^
**I2**	**2.07** (0.09) ^a*^	**1.71** (0.12) ^a,c^
**I3**	**2.09** (0.15) ^a*^	**1.65** (0.11) ^a,d^
**I4**	**2.60** (0.62) ^a*^	**1.58** (0.09) ^a,d^
**I5**	**0.43** (0.08) ^b*^	**0.50** (0.06) ^e^
**I6**	**1.45** (0.09) ^c*^	**0.82** (0.12) ^f^
**TC**	**2.12** (0.10) ^a^	**1.93** (0.19) ^c^
**SP**	**2.09** (0.11) ^a*^	**1.44** (0.06) ^b, d^

Within each column; different superscript letters indicate significant differences between the groups (P < 0.05). Within each row asterisk indicate significant differences between the paired groups (P < 0.05).

**Table 2.** Mean (SD) values of viscosity (MPa.s) of different resin composites at 23 °C and 37 °C.

**Group**	**Viscosity at 23 °C Mean (**SD**)**	**Viscosity at 37 °C Mean (**SD**)**
**I1**	**0.55** (0.27) ^a, d, e*^	**0.04** (0.01) ^a^
**I2**	**1.52** (0.20) ^b*^	**0.27** (0.05) ^b^
**I3**	**1.42** (0.34) ^b*^	**0.52** (0.14) ^b, c^
**I4**	**2.60** (0.62) ^b,c*^	**0.48** (0.13) ^b, c^
**I5**	**0.02** (0.01) ^d*^	**0.003** (0.001) ^d^
**I6**	**0.09** (0.02) ^e*^	**0.01** (0.002) ^d^
**TC**	**2.88** (0.61) ^c*^	**0.63** (0.10) ^c^
**SP**	**0.27** (0.06) ^a*^	**0.04** (0.01) ^a^

Within each column; different superscript letters indicate significant differences between the groups (P < 0.05). Within each row asterisk indicate significant differences between the paired groups (P < 0.05).

**Table 3.** Composition of resin composites used in the study.

**Resin-Composite**	**Filler Particles (Ground Glass [Ba-Al-B-silicate glass])**				**Matrix**
	**Shape**	**Size (nm)**	**Wt%**	**Vol%**	
**I1**	Irregular	450	76.4	56.7	BisGMA, UDMA, TEGDMA
**I2**	Irregular	700	76.4	56.7	
**I3**	Irregular	1000	76.4	56.7	
**I4**	Irregular	1500	76.4	56.7	
**I5**	Irregular	450, 1000 (1:3)	76.4	56.7	
**I6**	Irregular	450, 700 &1500 (1:1:3)	76.4	56.7	
**SP**	Spherical	100	72.4	56.7	
**TC**	Irregular& Spherical	40, 200 &1000	79	60	
**Lot:** C49490					
